# Flow units as dynamic defects in metallic glassy materials

**DOI:** 10.1093/nsr/nwy084

**Published:** 2018-08-24

**Authors:** Zheng Wang, Wei-Hua Wang

**Affiliations:** Institute of Physics, Chinese Academy of Sciences, Beijing 100190, China

**Keywords:** metallic glass, flow unit, flow, glass transition, mechanical deformation, property optimization

## Abstract

In a crystalline material, structural defects such as dislocations or twins are well defined and largely determine the mechanical and other properties of the material. For metallic glass (MG) with unique properties in the absence of a long-range lattice, intensive efforts have focused on the search for similar ‘defects’. The primary objective has been the elucidation of the flow mechanism of MGs. However, their atomistic mechanism of mechanical deformation and atomic flow response to stress, temperature, and failure, have proven to be challenging. In this paper, we briefly review the state-of-the-art studies on the dynamic defects in metallic glasses from the perspective of flow units. The characteristics, activation and evolution processes of flow units as well as their correlation with mechanical properties, including plasticity, strength, fracture, and dynamic relaxation, are introduced. We show that flow units that are similar to structural defects such as dislocations are crucial in the optimization and design of metallic glassy materials via the thermal, mechanical and high-pressure tailoring of these units. In this report, the relevant issues and open questions with regard to the flow unit model are also introduced and discussed.

## INTRODUCTION

An in-depth understanding of the structure–property relationship is a central concern in materials science. This interdependence is well understood in crystalline materials by controlling structural defects such as dislocations or twins [1–3]. These defects can be easily identified as broken long-range atomic order using a conventional transmission electron microscopy, and can be altered and manipulated during materials processing [3]. The discovery of these structural defects and the development of related theories not only explained many enduring mysteries, including the microscopic interpretation of plasticity and work-hardening in crystals, but also laid the foundation for designing new materials, which led to numerous successful applications in the last half-century [3,4]. In contrast, glassy materials or so-called amorphous materials including oxide glasses, which are regarded as the oldest artificial materials, most natural or synthetic polymers, rubbers and metallic glassy materials, lack a discernible periodic microstructure [5]. The common approach to fabricating glass is by quenching of a high-temperature liquid. Atoms or molecules in glass-forming liquids cannot rearrange into crystal lattice positions due to their thermal and kinetic limits, in contrast to crystalline materials. Liquid-like atomic structures in glassy materials invariably display maze-like patterns without any long-range order, which makes conceptualization and modelization very challenging [6]. As a result of the non-unique disordered microstructure, no conventional structural defects, such as dislocations in crystals, can be observed or identified in glasses. Therefore, glasses were cursorily treated as homogeneous, isotropic and defectless solids for a long time in history. However, with the development of experimental techniques and a deeper understanding of the amorphous state, it is now unambiguous that glassy materials are intrinsically spatially and temporally heterogeneous, and that this behavior is more obvious under external stimulus [7,8]. The discovery of locally short-range order or medium-range order in amorphous medium and even fractal connections also indicates the diversity of different regions in glasses [9–13]. These recent results all imply the existence of some ‘defect’ regions in glasses, which are not simply distinct in structure but reflect the distributed feature of dynamics. If such ‘defects’ from the heterogeneity can be distinguished, observed and further manipulated, new connections between the structure, dynamics, properties and even glass transition may possibly be established, which is long desired in this field.

Among all glassy systems, metallic glass (MG), which was first fabricated in a lab in the 1960s [14], possesses a simple atomic structure without motion from internal degrees of freedom. It is, therefore, a good model material for studying general issues in disordered systems. In addition, the superior properties, such as high strength, high toughness, high elasticity and premier soft magnetic performance, implies that MGs are possibly high-performance alternatives to many traditional crystalline alloys [15,16]. The low processing temperatures for MGs, which occurs near the glass transition temperature *T*_g_ (∼2/3 *T*_m_, which corresponds to a difference of hundreds of degrees for most alloys) can result in significant energy savings and facilitate a more environmentally friendly fabrication process [17]. However, the limited plasticity and even catastrophic failure at low temperatures hinder the wide application of these materials [18,19]. Even though a few MG compositions with good plasticity performance have been identified by luck among thousands of possible combinations in trial-and-error processes, and several processing methods have been successfully introduced, the atomistic mechanism is still unclear. Furthermore, the establishment of low-temperature brittleness with flowing behavior close to *T*_g_ is challenging, and requires a complete robust understanding of these processes.

In the last few years, dynamic ‘defects’ described as flow units have been observed in MGs and a theoretical framework was developed that defines these units and offers guidance on how to manipulate them, based on a series of robust experimental works. This theoretical perspective of flow units not only explains many important experimental phenomena of glasses, but also initially builds a unique structure–dynamics–property triangle relation for amorphous materials, which emphasizes the critical role of time-related characteristics in glasses. Herein, we outline an extensive review on the flow unit perspective to obtain a better understanding of this concept, since it is a newly proposed idea and is still in the process of development, although it is critically important to both the material science and physics fields. We begin this review with a historical background of classic models and the state-of-the-art studies on heterogeneities and dynamics in metallic glass field, which sparked the flow unit perspective. Then, the definition and evidence for flow units, including the constitutive equations, are presented. Furthermore, the activation mechanism of flow units and the correlation with changes in their properties under the application of external fields are summarized. The roles of flow units in the optimization of the properties of MGs are also introduced. The review ends with a discussion of outstanding issues and an examination of the future outlook of these materials.

## HISTORICAL BACKGROUND AND CURRENT CHALLENGES

For MGs, the objectives of understanding and controlling their mechanical behavior have attracted significant interest and are important considerations for potential applications. Intensive studies on the mechanical mechanism were conducted shortly after the discovery of MGs [20–22]. Several ingenious models were proposed in the late 1970s, including the widely utilized free-volume model [23] and the shear transformation zone (STZ) model [24]. These models could practically describe the mechanical behavior of MGs below *T*_g_, in spite of the limited size of the earliest MG samples [14]. Considering the inadequate knowledge of MGs at that time, all these models lack a connection with the microscopic structure and other intrinsic characterizations in real samples. The development of bulk MGs since the 1990s [25–27] offered the possibility for more precise measurements using cutting-edge testing technologies. This was not possible for micro-sized ribbon samples. Meanwhile, the rapid growth in computational power also introduced deeper insights in the form of simulations. As a result, more details of structure, dynamics and relaxation properties of MGs were uncovered in the last decade. It was discovered that the structure of MGs must be treated not simply as homogeneous but rather as heterogeneous in both the space and time domains [28,29]. Moreover, additional relaxation processes apart from the dominant α-relaxation were also observed in MGs [30–32], indicating the divergence of dynamics and the complexity of local structure. Local density and chemical fluctuations, in addition to short-range interactions, were found to be more important than ever anticipated, resulting in failure of the assumptions of mean-field theory in some cases [11,12,29]. Recent studies further revealed the existence of nanoscale liquid-like sites in the glassy state [33–35], which could be closely related to the viscoelastic and plastic flow behaviors in MGs. All these nontrivial advances made in the last ten years not only push the boundary of the understanding of MGs, but also challenge the basis of classic models. The flow unit perspective, which is developed based on the spatial and temporal heterogeneities and time-related dynamic features, is predicated on classic models and makes attempts to bridge the gap between theory and experiments.

### Classic deformation models of metallic glasses

#### Free-volume model

It has long been recognized that the difference in the volume expansion between a liquid and a glass is due to the excess volume [36]. The idea of the ‘free-volume model’ was first established by Cohen and Turnbull [37–39] to account for diffusive transport in liquids and glass transition. The free volume is defined as the empty space around atoms that can be redistributed in the system without a change in the energy. It has a constant value in the glassy state [22]. Similar to a vacancy in the lattice, a critical free-volume value *υ^∗^* is required to rearrange a particular atom. Later, in 1977, Spaepen [23] extended this model to explain the steady-state inhomogeneous flow behavior in MGs. A series of individual atomic jumps are assumed to lead to macroscopic flow. An atom can be squeezed into a neighboring smaller hole and more free volume will be generated by applying a sufficiently high stress on the sample. Such a stress-driven free-volume creation process is similar to the dilatation of grains of sand caused by the weight of a man walking on the beach [18]. At low temperatures, the diffusion-induced free-volume annihilation rate is low; therefore, plastic deformation and shear localization are expected to primarily occur on shear regions where more free volume is created.

The free-volume model can successfully explain the glass transition and mechanical performance of MGs with a simple and clear physical picture. However, the single-atom jump assumption has been found to be too simplified to reflect the real collective flow processes in glasses, where a large number of atoms are always involved [40]. Besides, the fraction of free volume in MGs is only approximately 2%. The notion that only a few ‘defects’ can carry the entire load of deformation sounds unrealistic [41]. In contrast, recent experiments show that almost a quarter of the total number of atoms are involved in anelastic behavior under load [33]. Plasticity initiates not only from low-density free-volume-rich regions but also from extremely dense regions with anti-free-volume [41]. This result means that free volume alone cannot explain the origin of plasticity in metallic glasses. Furthermore, the free-volume model cannot explain the divergence of dynamics in glasses, which is understandable since the concept was introduced subsequent to this model.

#### Shear transformation zone model

In 1977, Argon [24] introduced the shear transformation zone (STZ) model to interpret the plastic deformation in MGs, inspired by sheared soap bubble experiments. Localized clusters of atoms are conceived to be five atom diameters in Argon's original paper [24] since flow defects can undergo irreversible rearrangements and accommodate flow in MGs under applied stress. The activation dilatation is produced by pushing apart surrounding atoms along the activation path rather than squeezing a single atom, as in the free-volume model. The STZ model was further developed by introducing an orientational degree of freedom [42], and the STZs are considered to transform from one orientation to another and contribute to the irreversible shear deformation [43]. In a nonequilibrium system, the STZ density is proportional to exp(−1/χ), where the reduced effective temperature χ = *k*_B_*T*_eff_/*E*_z_ [44]. The effective temperature *T*_eff_ is the inverse of the derivative of the configurational entropy with respect to configurational energy [44]. Therefore, such a relation is a direct analog of the free-volume formula by replacing the reduced free volume with χ [45]. The success of the STZ model is evident in both its ability to reproduce the macroscopic stress–strain relation and to explain the origin of the strain localization that leads to the formation of shear bands [43]. Based on the potential energy landscapes (PEL) and Frenkel's dislocation theory, Johnson and Samwer proposed a cooperative shearing model (CSM) for STZs [46], and a universal (*T*/*T*_g_)^2/3^ law was then obtained for the flow stress of MGs. The CSM model provides quantitative insight into the atomic-scale mechanisms of the plasticity of MGs at low temperatures. They later correlate the isolated STZ transitions confined within the elastic matrix with underlying configurational hopping mechanisms [47]. This is distinct from Falk and Langer's STZ but more similar to the activation of flow units, as discussed later. Based on studies on assumptions from the CSM model, the volume of STZs including 200−700 atoms was experimentally determined [48]. The STZ model possibly ignores the structural origin in real materials, and the creation, shear transition, and annihilation of STZs are considered as noise-activated processes [43]. This is not in agreement with the recent experimental observation of spatial and temporal heterogeneities of MGs, as discussed in the next section. It is worth noting that there are several variations with distinct definitions from the original STZ paper. Here we only take the strict definition from Falk and Langer [43] as it is the most used one. In a general sense, the flow unit may also be regarded as a kind of STZ since both refer to the fundamental deformation unit in metallic glasses.

### Spatial and temporal heterogeneities of metallic glasses

It has long been recognized that supercooled liquids are dynamically heterogeneous, and the dynamics in some regions can be orders of magnitude faster than the dynamics in other regions [7,8]. Glasses are normally quenched from liquids and naturally inherit the disordered structure and dynamic information from the corresponding liquid. It has been theoretically postulated and experimentally observed that MGs exhibit both spatial and temporal heterogeneities, compared to their crystalline counterparts [28,29,49]. By using atomic force acoustic microscopy (AFAM), a Gauss-like distribution of local indentation modulus on a scale below 10 nm in a PdCuSi MG sample was discovered, with a variation up to 33%. Meanwhile, crystallized PdCuSi showed a variation that is 10–30 times smaller [29]. A wide variety of meta-basins in 3 N dimensions of the PEL for MGs in contrast to a crystalline ground state is therefore expected. Nanoscale mechanical heterogeneity of ZrCuNiAl MG films was also revealed and characterized by taking advantage of dynamic atomic force microscopy [28]. Low and high phase shift domains were distinguished in the MG sample, with an average viscosity difference ∼10%. Those high phase shift domains perform in a more ‘liquid-like’ way because of their relatively low viscosity and elastic modulus. The characteristic length of the viscoelastic heterogeneity was found to be ∼2 nm and originates from the intrinsic material behavior. By utilizing ultrasonic annealing of PdNiCuP MG below *T*_g_, partially crystallized samples with a hierarchical microstructure are obtained. Moreover, weakly bonded regions with a high atomic mobility will first crystallize after being stimulated by ultrasonic vibrations [50]. Such heterogeneous structure of MGs is further suspected to relate to secondary β-relaxation. More evidence of the heterogeneous dynamics in MGs is from the stretched exponential form of the distribution of relaxation times, and the superposition of the spatial domains with distinct dynamic characteristics can be reflected in the frequency or temperature domain relaxation spectrum [51]. The spectrum is normally described using the Havriliak–Negami (HN) function or, alternatively, with the Fourier transform of the Kohlrausch–Williams–Watts (KWW) function [52]. Both the HN and KWW functions can be used to capture the features of non-exponential processes, where only one fractional exponent *β*_KWW_ is used in the KWW function instead of the two shape parameters in the HN function. For simplicity, we will only discuss the meaning of *β*_KWW_ and its correlation with heterogeneous dynamics since the conclusions for the HN fitting parameters are basically the same [8]. *β*_KWW_ normally has a value close to 1 at high temperatures. This implies a single exponential relaxation process, and the value decreases near *T*_g_ [7]. A *β*_KWW_ smaller than 1 indicates non-exponential relaxation and a reasonable fundamental explanation is that relaxations within each local region could be exponential but the spatial relaxation times vary significantly, leading to a non-exponentiality regarding the ensemble average [7,8]. For most MGs, the fractional exponent *β*_KWW_ of the KWW function is between 0.4 and 0.8 [30,31,53–55], indicating a broad distribution associated with heterogeneous dynamics. Therefore, some high-mobility regions with short relaxation times have a liquid-like behavior at the experimental timescale and possibly act as a fundamental deformation unit of the flow unit, as introduced in the next section. These advances in the heterogeneous nature of MGs in both structure and dynamics were mainly made in the last decade and challenged the basis of the classic models proposed half a century ago.

### Dynamic mechanical behaviors of metallic glasses

In liquids, the dynamics is characterized by diffusivity and viscosity, where the two statistics are in inverse proportion and follow the Stokes–Einstein relation at high temperatures [56]. A single relaxation mode is believed to exist in high-temperature liquids. Yet below a critical temperature ∼1.2*T*_g_, the inverse relationship between diffusivity and viscosity breaks down and long-range atomic transport starts to freeze in. One relaxation peak also splits into primary (α) and secondary (β) relaxation in the supercooled regime [56,57]. The α-relaxation follows a non-Arrhenius behavior, which means that its relaxation time increases speedily at a low temperature, and finally disappears at the experimental timescale at *T_g_*. The β-relaxation, which displays Arrhenius behavior, continues below *T*_g_ and therefore becomes the principal source of dynamics in the glassy state. Considering that there are no intramolecular degrees of freedom in MGs [58], β-relaxation in MGs is the analogue of Johari–Goldstein relaxation in non-metallic glasses [32,55,58]. Given that the dielectric spectroscopy method is non-feasible for conductive MGs, dynamical mechanical analysis (DMA) is widely applied to study the relaxation dynamics in MGs [30,31,53,59]. One advantage of using the DMA method is the high sensitivity of the loss modulus *E*″ spectra with respect to dynamic defects that are rooted in the atomic configuration and have been proven to be useful for finding defects in crystalline solids [60,61]. For MGs, there are three different kinds of secondary relaxation forms: (1) A peak shape that is often found in RE-based MGs (RE = rare earth); (2) A shoulder shape that is often found in Pd-based MGs; (3) An excess wing that is often found in Zr- and Cu-based MGs. The composition/chemical effects on β-relaxation behavior have been explained. This included the consideration of the enthalpies of mixing between constituent atoms [62]. Recent progress in regard to the discovery of more robust MG systems with obvious secondary relaxation peaks apart from the primary relaxation [31,63–65] has been beneficial to further studies on dynamic defects and their correlation with mechanical performance in glassy states. Very recent experimental results show that the β-relaxation can be suppressed in ultrastable glasses [66], which can be considered as a glassy state very close to ideal glass. The divergence of relaxation is inadequately addressed by previous models, although its importance in understanding glassy properties is clear. This is the inspiration for the proposal regarding the flow unit.

## FLOW UNIT PERSPECTIVE IN METALLIC GLASSES

### Definition of flow units

**Figure 1.**
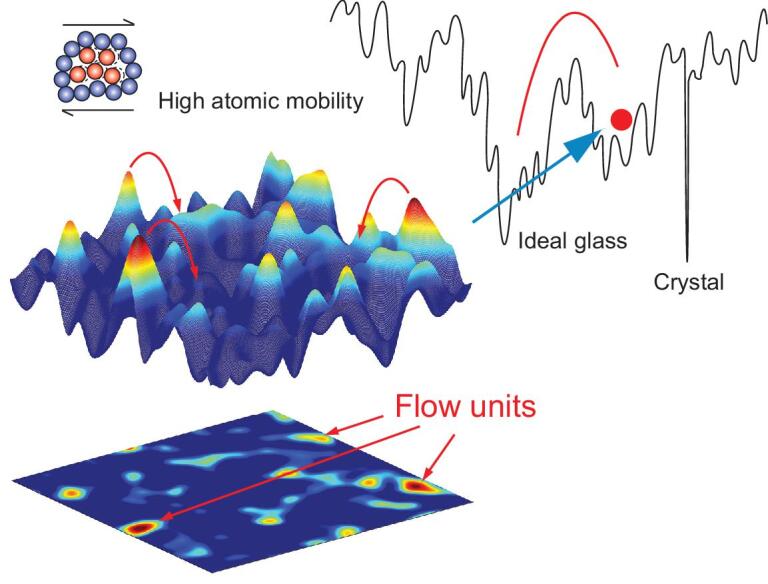
Flow units are fast dynamic regions with high/unstable energy states in structure and are responsible for the time-dependent properties in a glassy state.

The existence of spatially distributed dynamic ‘defects’ in MGs was considered a long time ago but lacked solid experimental evidence. For example, Cohen and Grest [67] speculated that an inhomogeneous free-volume density distribution in glasses could result in disparate solid-like and liquid-like cells. The concept of flow defects was also used in Argon's original paper of the STZ model [24]. However, these dynamical flow defects, which are closely related to the heterogeneous nature of MGs, were not experimentally proved and characterized until recently [33–35]. The ‘flow units’ perspective is proposed to describe the newly discovered heterogeneous nature of MGs. Flow units are regions with faster dynamics that perform like a liquid compared with the solid-like matrix at an experimental timescale. Liquids under shear will follow the relation *σ* = *G*(*t*)*ε*, where the shear modulus *G*(*t*) is a decaying function with time *t* [68]. Liquids can also be elastic only on timescales shorter than their intrinsic relaxation time [69]. The experimental or observing timescale is normally of the order of 100 s, which is defined as the separation time between glass and liquid [56,69]. Therefore, the flow units are the regions that mainly contribute to the viscoelastic feature of MGs. Furthermore, internal friction during the flowing process of atomic-scale flow units could dissipate the energy after loading through jumping to a lower-energy minimum, in a similar role to dislocation sliding in a crystal. Figure 1 shows the flow units and their spatial distribution at low temperatures in an MG, where loose atomic packing, a low localized modulus and strength, small effective viscosity, high atomic mobility and high/unstable localized potential energy state are their main characteristics. The collaborated atomic motions within the flow unit will be reflected as β-relaxation. The average effective size of flow units, which normally contain hundreds of atoms and occupy a volume of several cubic nanometers in space, differs for diverse compositions and at different energy states. Since the dynamics are governed by these fast relaxed flow unit regions in the glassy state, a real MG sample can be simplified as a combination of an ideal elastic matrix and distributed flow units, in an attempt to understand the underlying mechanism of mechanical behavior. The ideal glassy matrix is solid-like and behaves elasticity, and the flow units are more like a liquid with a behavior that is time-dependent at the experimental timescale.

### Experimental evidence for flow units

The heterogeneous nature of MGs, especially the wide distribution of relaxation time composed of diverse mobility regions, is strong justification for the rationale for the existing flow unit regions with fast dynamics as presented in the section entitled ‘Spatial and temporal heterogeneities of metallic glasses’. Recently, the observation of viscoelastic behavior in the apparent elastic regime of MGs by both micro- and macroscopic experiments further validated the flow unit perspective [33–35,70–72]. From the *in situ* tensile creep experiments on Zr-based Vit105 bulk MG at 300 K, which was analyzed using synchrotron X-ray diffraction [33], an average of 24% of the total strain is found to be viscoelastic, which means that almost a quarter of the volume is occupied by flow-unit-like regions under stress in this MG. Besides, from cyclic loading tests on micropillars [34], ribbon shapes [35] or bulk-sized MG samples [70–72], mechanical hysteresis loops can be observed in all these experiments irrespective of whether the sample was subjected to compression, tension or shear loading. The phase lag between the two signals reflects the characteristic viscoelastic property [2], and the lower effective viscosity or higher fraction of the liquid-like portion in the whole sample under a faster loading–unloading deformation rate introduces a larger phase lag or more obvious hysteresis loop. Therefore, constitutive equations can be determined based on the viscoelastic feature of the MG based on the flow unit perspective. These recent experimental observations reveal that the flow unit perspective based on spatial and temporal heterogeneities is better suited to the investigation of the features and properties of MGs.

### Constitutive equations of flow units

**Figure 2.**
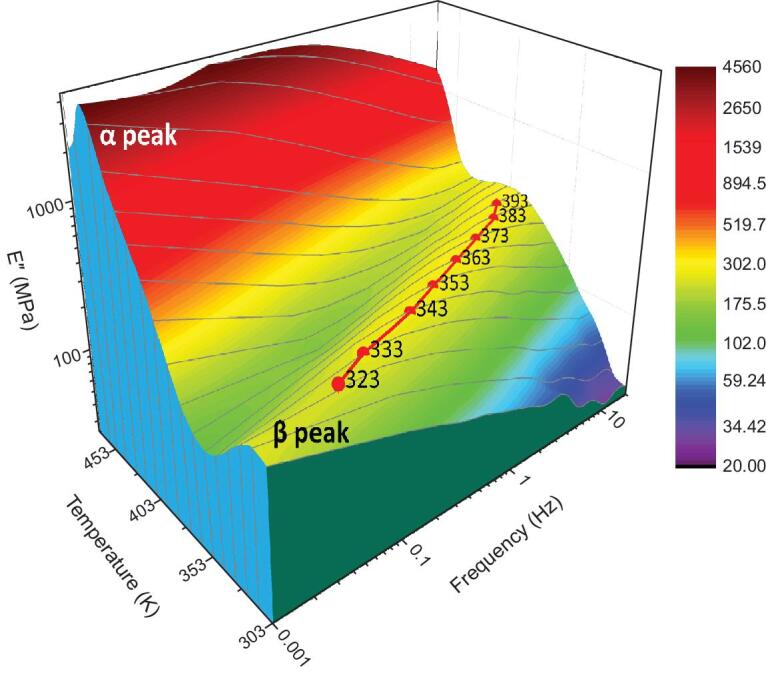
3D dynamic mechanical relaxation map. Adapted with permission from [73], Copyright 2014, Nature Publishing Group.

The dynamical mechanical method is an effective approach for studying the properties and evolution of flow units in MGs. The temperature/frequency-dependent loss modulus *E*″ of the dynamical mechanical spectrum (DMS) quantifies the viscoelastic properties of a material. This parameter can be calculated from the phase lag between the stress and strain. As an example, a 3D loss modulus *E*″ map measured on LaNiAl bulk MG is shown in Fig. 2 [73]. All the dynamical information for the tested MG can be captured via the DMS within a detectable temperature and frequency range. The governing dynamic process in the glassy state is β-relaxation, which can only stem from high-mobility, liquid-like flow units. The strength and distribution of β-relaxation is indicative of the characteristic of the flow unit regions, and a 30% difference in the fraction of flow unit regions is found in as-cast La-based and CuZr-based MGs [35]. The change of β-relaxation with temperature as shown in Fig. 2 also reflects the evolution of the flow units as they approach *T*_g_ [73,74].

The cyclic loading–unloading or analogous loading–holding–unloading deformation profiles performed for various types of MGs show an obvious mechanical hysteresis loop in the stress–strain curve, which reveals the underlying mechanism of the viscoelastic behavior [35]. To characterize the activated flow units responsible for the viscoelastic behavior, a three-parameter mechanical model based on the Voigt model was proposed to simulate the deformation process [35]. A viscous dashpot in series with a spring representing the flow units is placed in parallel to an elastic spring that represents the matrix. The two components work synergistically to maintain the applied stress. The governing equation of the three-parameter model under tension is then expressed as [35]

**Figure 3.**
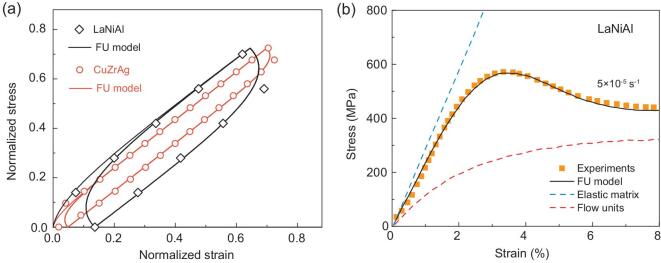
Experimental data and simulated curves based on the flow unit perspective for (a) mechanical hysteresis loop and (b) stress–strain curve of MGs. Data taken from [35] and [75].

(1) \begin{equation*} ({E_1} + {E_2})\eta \dot{\varepsilon } + {E_1}{E_2}\varepsilon = \eta \dot{\sigma } + {E_2}\sigma \end{equation*}

where *σ* is the applied stress and *ε* is the resulting strain; *E*_1_ and *E*_2_ represent the Young's modulus of the matrix and the outlayer of the flow units, respectively; and *η* is the average effective viscosity of the flow units. The main feature of the hysteresis curves can be captured by a model with the same input signal as the experiments, as shown in Fig. 3. The simulated effective viscosity of the flow units in the LaNiAl and CuZrAg MGs samples are ∼1.5 and ∼4 GPa, respectively [35]. Quasi-static cyclic compression results on bulk MGs can also be simulated using this three-parameter model, which shows the activated fraction change of flow units for Zr-based and Mg-based MGs [71]. The difference in the flow unit characteristics for various MG systems is not only related to their viscoelastic behavior, but also leads to distinct macroscopic mechanical behavior.

Typical stress–strain curves, especially those curves that exhibit homogeneous flowing behavior, can also be simulated using our flow unit perspective based on the three-parameter mechanical equation. The total stress is sustained by both the elastic matrix and the flow units according to the following equation [75]:

(2) \begin{equation*} \sigma = (1 - {c_{{\rm{FU}}}}){\sigma _{\rm{E}}} + {c_{{\rm{FU}}}}{\sigma _{{\rm{FU}}}} \end{equation*}

where *c*_FU_ is the fraction of flow units. *σ*_E_ is the partial stress contributed by the elastic matrix as ${\sigma _{\rm{E}}} = {{\rm{E}}_1}\varepsilon $; *σ*_FU_ is the partial stress contributed by the flow units as ${\sigma _{{\rm{FU}}}} = {\sigma _{\rm{s}}}(1 - \exp( - {E_2}\varepsilon /{\sigma _{\rm{s}}}))$, where *σ*_s_ is the stable flowing stress. The fraction evolution of the flow units under different strain rates can be described by a rate equation [75]:

(3) \begin{equation*} d{c_{{\rm{FU}}}}/dt = - k({c_{{\rm{FU}}}} - c(0))({c_{{\rm{FU}}}} - c(\infty )) \end{equation*}

where *c*(0) and *c*(1) represent the lower and upper fraction limits, respectively. Simulated stress–strain curves with different strain rates are all in good agreement with the experimental data measured on the LaNiAl bulk MG at 413 K (∼0.9*T*_g_) [75], and an example is shown in Fig. 3.

Further, considering the activation of flow units as hopping events across inherent structures from the PEL, the increase of the total energy of the system under external stress can be derived as [72]:

(4) \begin{equation*} \dot{\varepsilon } + 2\omega {e^{ - \frac{{{E_{{\rm{FU}}}}}}{{kT}}}}\varepsilon = \frac{{\dot{\sigma }}}{\mu } + 2\omega {e^{ - \frac{{{E_{{\rm{FU}}}}}}{{kT}}}} \left(\frac{{\beta \Omega }}{{kT}} + \frac{1}{\mu }\right)\sigma \end{equation*}

where ω, *E*_FU_ and Ω are the attempt frequency, activation energy and activation volume for the flow units, respectively. *μ* can be treated as the modulus of an ideal glass without flow units. This equation has the same form as the constitutive relation from the three-parameter viscoelastic model in Equation 1, but under the shear deformation condition. The equation has the form [72]

(5) \begin{equation*} \dot{\varepsilon } {+} \frac{{{G_{\rm{I}}}{G_{{\rm{II}}}}}}{{\eta ({G_{\rm{I}}} + {G_{{\rm{II}}}})}}\varepsilon = \frac{{\dot{\sigma }}}{{{G_{\rm{I}}} + {G_{{\rm{II}}}}}} + \frac{{{G_{{\rm{II}}}}}}{{\eta ({G_{\rm{I}}} {+} {G_{{\rm{II}}}})}}\sigma \end{equation*}

Here, *G*_I_ represents the quasi-static shear modulus of the tested sample and *G*_II_ represents the shear modulus contribution of the flow units. From Equations 4 and 5, one gets [72]

(6) \begin{equation*} {G_{\rm{I}}} = \frac{\mu }{{1 + \alpha }}\end{equation*}

and

(7) \begin{equation*} {G_{{\rm{II}}}} = \frac{{\alpha \mu }}{{1 + \alpha }}\end{equation*}

where $\alpha = \beta \Omega \mu /(kT)$ is a factor that takes into consideration the total effect of the aggregated flow unit regions. A simple relation between *G*_I_ and *G*_II_ can be derived as [72]

(8) \begin{equation*} {G_{\rm{I}}} = \mu - {G_{{\rm{II}}}}\end{equation*}

This indicates that the shear modulus of a real MG sample can be obtained by subtracting the flow-unit-contributed shear modulus from the shear modulus of an ideal glass state. From the above equations, it is seen that, if *α* approaches infinity, *G*_I_ will be zero, and the three-parameter viscoelastic model will reduce to a Maxwell model, which is commonly adopted for supercooled liquids [69]. If *α* approaches zero, the entire sample will be in an ideal glass state without the existence of any flow units, which is supported by the absence of β-relaxation in ultrastable glasses [66]. Therefore, the factor *α* can be used to quantify the influence of flow units when considering the aforementioned two extreme conditions.

From the flow unit perspective, Equation 8 coincidentally has a similar form to another independent interstitialcy theory [76,77], where the main equation relating the shear modulus *G* of glasses can be written as [77]

(9) \begin{equation*}G = {G_0}\exp( - \alpha \beta {c_{{\rm{ID}}}})\end{equation*}

where *G* is the shear modulus of glass, which is the same as *G*_I_, *G*_0_ is the shear modulus of the defectless crystal, which is close to *μ*, *α* is a dimensionless constant almost equal to 1, *β* is the interstitialcy shear susceptibility and *c*_ID_ is the interstitialcy defect concentration. For small *βc*_ID_ at room temperature, Equation 9 can be expanded into a series as $G = {G_0} - \beta {c_{{\rm{ID}}}}{G_0}$, which has an identical form to the relation for the flow unit represented by Equation 8. Such a similarity may imply that the flow units can also be viewed as types of ‘defects’ in MGs.

Therefore, the main features of flow units can be summarized as follows: (1) A normal glass can be treated as exhibiting a combination of a glass matrix elastic property and distributed flow units with a time-dependent viscous property; (2) The elastic matrix can be modeled using a series of mechanical springs with similar modulus, while each flow unit can be modeled as a mechanical dashpot and a distribution of all flow units exists. In some cases, an average relaxation time or viscosity can be used to reflect the overall contribution of the flow units and a three-parameter mechanical model can be deduced; (3) An energy barrier needs to be overcome to activate flow units, similar to the hopping event across inherent structures from the PEL; (4) The activation of flow units can be stimulated by either temperature or stress or both; the activation process is discussed in detail in the section entitled ‘Properties of flow units and their evolution under external fields’; (5) flow units are always isolated and stochastically activated under small external stimulation. The activated flow unit density increases with intensive stimulation until a critical percolation value is reached.

## PROPERTIES OF FLOW UNITS AND THEIR EVOLUTION UNDER EXTERNAL FIELDS

### Correlation between flow units and dynamic heterogeneity

**Figure 4.**
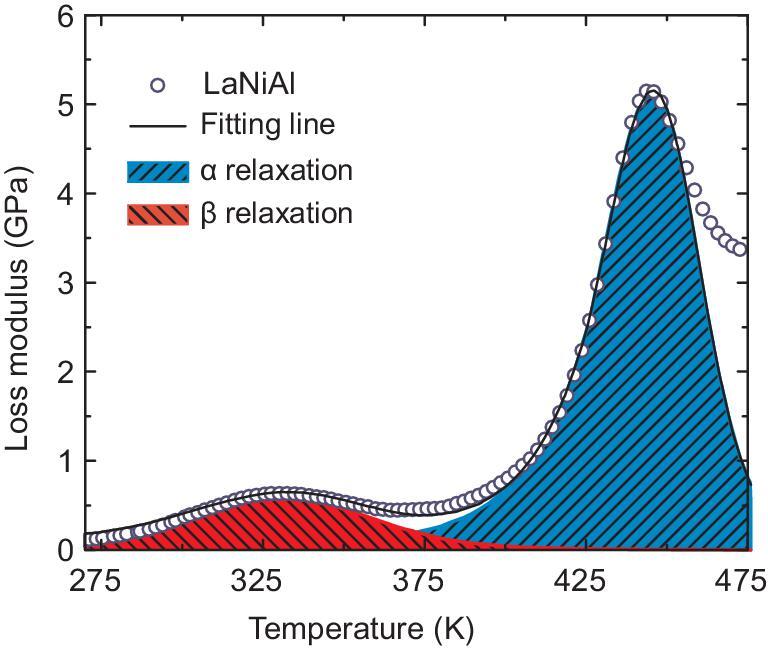
Temperature-dependent loss modulus measured at 1 Hz and corresponding KWW fitting. Adapted with permission from [35], Copyright 2012, American Institute of Physics.

The dynamic heterogeneity in MGs is reflected by the stretched exponential relaxation behavior. Using the KWW function to fit the α-relaxation peak, a fitting parameter *β*_KWW_ is determined, which is related to the distribution of relaxation times. Normally a smaller *β*_KWW_ value indicates a wider dynamic heterogeneity distribution, and *vice versa* [7,8,78]. One fitting example is shown in Fig. 4 for a La-based glass-forming composition with a pronounced β-relaxation peak [35]. A uniform *β*_KWW_ can be used to capture the distribution shape of either α- or β-relaxation peaks from isochronal and isothermal DMS [73], indicating an intrinsic link between these two relaxation processes, or even fractal dynamics at different timescales. The possible fractal dynamics nature of the flow units may be related to a recently discovered avalanche behavior during deformation [79,80]. However, for most MG systems, a merging β-relaxation shoulder or wing hinders the process of accurate fitting to obtain *β*_KWW_, and a limited supercooled liquid range for certain poor glass formers will worsen the process further.

**Figure 5.**
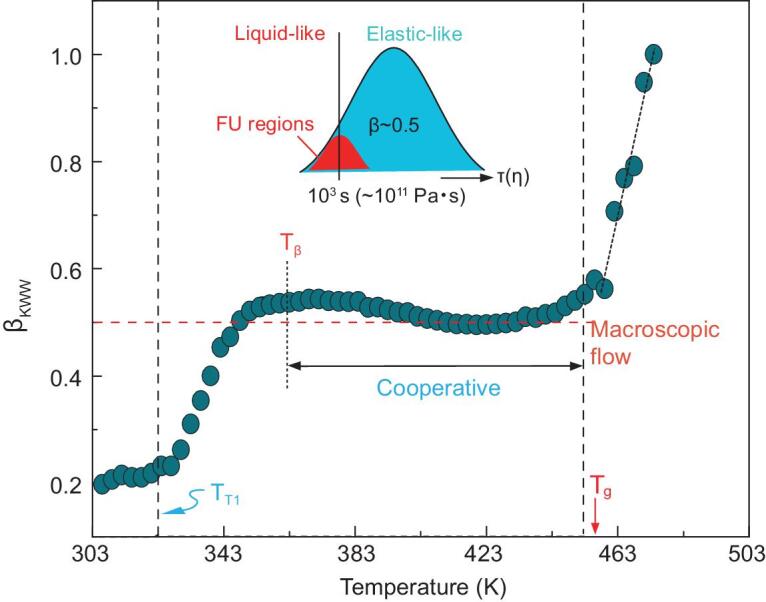
*β*_KWW_ values reflecting the change of dynamic heterogeneity with temperature. Data taken from [73].

An alternative way to obtain *β*_KWW_ is through stress relaxation, which is also useful in the study of the time-dependent behavior of flow units in MGs. During experiments, the stress change with time can be fitted using a KWW-type equation [73]:

(10) \begin{equation*} \sigma (t) = {\sigma _0}{\rm{exp}}{( - t/{\tau _{\rm{c}}})^{{\beta _{{\rm{KWW}}}}}} + {\sigma _{\rm{r}}}\end{equation*}

where *σ*_0_ is the initial stress, *σ*_r_ is the residual stress at a finite time and *τ*_c_ is the critical stress relaxation time. To obtain all the parameters that accurately reflect the characteristics of the entire sample, *σ*_r_ = 0 is taken until *T*_g_. Maintaining *σ*_r_ below 1 MPa is ideal for better fitting above *T*_g_ [73]. Assuming that the MG sample is ‘homogeneously’ disordered, the fitted *β*_KWW_ value should be kept constant at any measured temperature below *T*_g_, since the whole sample can be treated as an integral-like viscous liquid over an infinite time, and contributes to the stress decay. However, this assumption is overturned by experimental results, where *β*_KWW_ always deviates from the standard value at low temperature [73,81], as shown in Fig. 5. Compared to the standard *β*_KWW_ value of 0.5 for the tested sample, smaller fitted *β*_KWW_ values below *T*_β_ were observed, demonstrating an inhomogeneous activation of flow units. The reason for this deviation is that only parts of high-mobility flow units, with a distribution that is far from the global relaxation distribution of the sample (see inset of Fig. 5), were activated and exhibited time-dependent resistance against external loading stress at low temperature. Therefore, the stress relaxation curve cannot be properly fitted using the average global relaxation time and the standard *β*_KWW_ value, which describes the heterogeneity of the whole sample in this case. The experimental data can only be fitted with a reasonable standard *β*_KWW_ value by introducing an additional exponential function into Equation 10, which possesses a shorter relaxation time *τ*_FU_ from the flow unit perspective [73]. The results further confirm the connection between dynamic heterogeneity and the existence of flow units in MG systems. The normalized activation energy spectra *P*(*E*) of the flow units at different temperatures can also be calculated by using an extended Maxwell equation [82,83], showing a wide distribution as expected from the small *β*_KWW_ values. The rapid increase of the fitted *β*_KWW_ value above *T*_g_ is attributed to a shorter global mean relaxation time relative to the experimental waiting time. This suggests that the whole sample enters into a liquid state at the same order of experimental waiting time (∼10^3^ s) rather than at ‘infinite’ time (> 10^8^ s) for room-temperature glasses [73].

### Activation mechanisms of flow units

**Figure 6.**
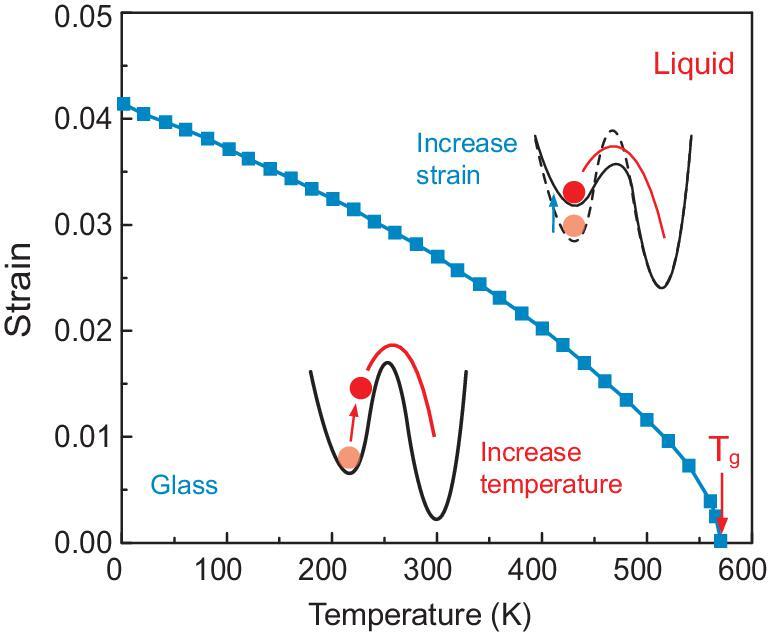
Diagram of glass transition in MGs achieved by increasing temperature or applying strain. Insets are illustrated mechanisms in the PEL. Data taken from [86].

As hidden dynamic ‘defects’ in MGs, flow units can demonstrate dynamical flowing behavior only after being activated by an external stimulation. Increasing the temperature and applying stress to MGs are two widely adopted approaches for activating flow units. An increase of the temperature will simultaneously increase the energy of each atom. Systems in a high-energy state have a higher probability to overcome the energy barrier into a new state, and the average relaxation time will decrease as a result. As regions with high mobility at unstable localized potential energy sites, flow units will naturally be activated with an increase in temperature. On the other hand, an external force will flatten the barrier between local energy minima and prompt a system jump to a lower-stress energy minimum [84]. Considering the heterogeneous nature of MGs and the stress concentration on soft regions, flow units are prone to start flowing after activation and even further aggregate to form shear bands along the force direction. Even though the mechanisms of activation associated with these two methods are not exactly same, the contribution to achieve a liquid-like behavior is shared by the temperature and applied stress. A relation between these two factors was revealed by *ab initio* molecular dynamics (MD) simulations as [85]:

(11) \begin{equation*} \frac{T}{{{T_0}(\eta )}} + {\left(\frac{\sigma }{{{\sigma _0}(\eta )}}\right)^2} = 1\end{equation*}

where $T/{T_0}(\eta )$ and $\sigma /{\sigma _0}(\eta )$ represent the normalized effect of temperature and the applied stress, respectively. By modeling the flow units as liquid-like quasi-phases embedded in a solid-like glassy substrate, a diagram demonstrating the temperature- and stress-induced glass transition based on the activation of flow units is obtained as shown in Fig. 6 [86]. This result is in agreement with Equation 11 based on MD simulations. Such ‘strain–temperature equivalence’-facilitated activation of flow units can also be observed by combining DMS with linear-heating stress relaxation experiments [87].

The activation energy *E*_FU_ of the flow units can be obtained from DMS measurements of the shift of the β-relaxation peaks, which ranges from 0.5−2 eV [88]. The critical volume of activated flow units is proportional to *E*_FU_ and can be calculated from [79]

(12) \begin{equation*} \Omega = {E_{{\rm{FU}}}}/(8/{\pi ^2})\gamma _{\rm{c}}^2\zeta G\end{equation*}

where *γ*_c_ ≈ 0.027 is the average elastic limit, *ζ* ≈ 3 is a correction factor and *G* is the shear modulus. The calculated volume for a typical MG systems varies from 2−8 nm^3^ and contains approximately 200 atoms for each activated flow unit [89]. Besides, based on direct observations of nanoscale PdSi droplets using a double-aberration-corrected scanning transmission electron microscope (Cs-STEM), it was determined that a critical size of 2.3 ± 0.1 nm was required to avoid nucleation [90]. Taking into account that the droplet is a half-sphere on a plate substrate, the critical amorphous volume should be approximately 10 nm^3^. As a high-energy and high-mobility unstable region similar to the droplet with a free surface, flow units with an activation volume less than 10 nm^3^ are reasonable. It is worth noting that the size of flow units should have a distribution from the dynamic heterogeneity and the boundary of a flow unit should also be time dependent. Here we only consider the average size of flow units with observing times close to their relaxation times for simplicity. Recently, Krausser *et al.* [91] and Wang *et al.* [92] predicted the deformation unit sizes from interatomic repulsion-controlled liquid dynamics, which is in agreement with the flow unit sizes determined from our calculation. This result may offer a deeper insight into the understanding of flow units based on atomic interaction and further confirm that the flow unit is intrinsic in MGs.

A recently discovered fast relaxation process termed fast β- or γ-relaxation can be activated at low temperatures [93,94]. The activation energy is around 0.5 eV and is insensitive to *T*_g_ [95]. This fast process could be intrinsic from the topological heterogeneity and may correspond to those most mobile regions of flow units. This result may indicate that some sub-flow units stay active at low temperatures when most flow unit regions become dormant. There is still a lot to be done to fully understand this fast process since it is newly found.

### Evolution processes of flow units under external temperature fields

**Figure 7.**
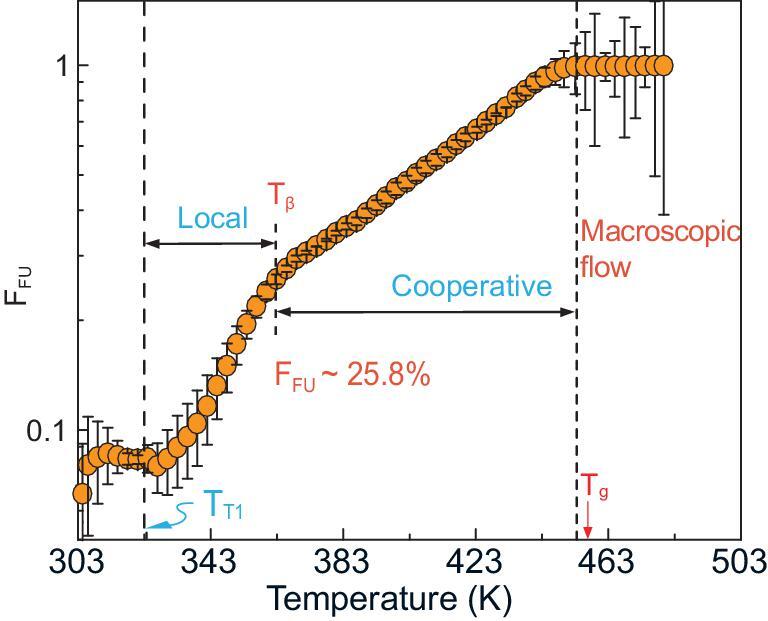
Evolution path of the fraction of the flow units *F*_FU_ with increasing temperature. Cooperative motions start after *F*_FU_ reaches a connectivity percolation threshold ∼ 0.25. Data taken from [73].

Nearly constant loss (NCL) was observed in the relaxation curves at low temperatures or in the high-frequency regime beyond β-relaxation in many glass-forming systems including MGs [96–99]. From the point of view of flow units, low-temperature NCL dynamics correspond to reversible atomic motion within the flow units and the system remains in the original local minimum on the PEL [100,101]. With an increase in the temperature, flow units begin to be activated, but initially in an isolated and stochastic way. Adjacent weakly bonded regions around the early-formed flow units will also gradually transform into a liquid-like state and the fraction of flow units will increase as the temperature continues to increase. This process can be facilitated under a simultaneous deformation. At this stage, localized plastic events start to occur and the yielding stress will decrease. There is no obvious global plasticity for brittle MGs or tensile plasticity for ductile MGs [73]. All flow units that originate from spatial heterogeneity are fully activated at temperatures above *T*_β_ and reach the threshold volume fraction of 0.25–0.3 of connectivity percolation for a 3D continuum system [102–105], as shown in Fig. 7. The percolation of flow units favors the formation of multiple shear bands and leads to a ductile deformation behavior. It is noted that the commencement of connectivity percolation of the flow units will not immediately change the rigid nature of the sample to external forces, especially at a low strain level. Such cooperative translational movements can be regarded as a kind of confined glass transition, leading to a sub-*T*_g_ endothermic peak termed the shadow glass transition [106,107]. When the temperature approaches *T*_g_, a heat capacity jump of 3R/2 caused by the additional translational freedom can be observed and a critical transition from broken-ergodic to ergodic of the energy landscape occurs. Additional translational freedom implies a rigidity percolation of the flow units, which causes the system to lose the ability to permanently sustain an applied stress [108,109]. The sample has a macroscopic flow behavior above *T*_g_ and the viscosity then becomes the dominating factor rather than the fraction of flow unit. This is because the average global relaxation time is shorter than the experimental waiting time and the whole sample can be treated as liquid-like [73]. A universal crossover phenomenon in various MGs [83] reflected by the sudden drop of yield strength close to *T*_g_ demonstrates that the flow unit description is valid for MGs.

### Evolution processes of flow units under external stress fields

**Figure 8.**
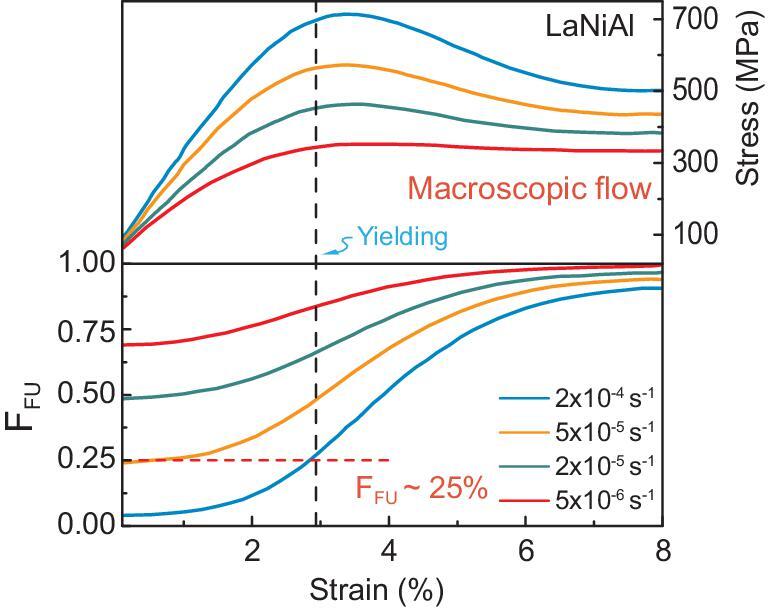
Evolution path of the fraction of the flow units *F*_FU_ at different strain rates. Macroscopic flow is achieved after *F*_FU_ reaches ∼ 0.25 and cooperative motions start. Data taken from [75].

External forces can also activate flow units. The stress relaxations observed under different strain status were measured for LaNiAl MGs at 370 K (∼0.75*T_g_*) to study the activation and evolution of flow units under strain [110]. A large stress drop after ∼1000 s of holding time was noticed for a higher applied strain. This implies that a larger fraction of sample was activated. The average relaxation time *τ*_FU_ and non-exponential parameter *β*_FU−KWW_ show a three-stage evolution upon increasing the applied strain [110], which has a similar trend to that observed under the influence of an enhanced temperature field. Most mobile parts of the flow units will first be activated under a small external strain. The holding time is even longer than the average relaxation time of these fast dynamics regions, leading to a large *β*_FU−KWW_ value close to 1. When more intrinsic flow units are activated, *β*_FU−KWW_ will drop back to its intrinsic value of approximately 0.5. In addition, *τ*_FU_ will increase up to an almost constant value, indicating that the activated fraction is the governing parameter to determine the mechanical behavior at this stage. Approaching yielding strain, the decrease of *β*_FU−KWW_ and the increase of *τ*_FU_ both demonstrate that parts of the matrix are also involved in the expressed behavior except for those intrinsic flow units. Cooperative movement of flow units and shear dilatation effects will easily result in the formation of shear bands and eventually lead to a breakdown under the strain level. Compression tests on a bulk sample of similar composition measured at 413 K (∼0.9*T*_g_) with lower strain rates are capable of achieving a stable flow at larger strain ranges [75]. Stress–strain curves can be simulated based on the flow unit perspective using Equations 2 and 3. The activated fractions of the flow units under different strain rates are obtained and shown in Fig. 8. Large fractions of the sample exhibit a liquid-like behavior when exposed to external stress fields with slower strain rates. The different strain rates can actually resale the cut-off position of the dynamics limit of flow units, and the slower strain rate with a longer observing time will push the dynamic limit to the slower side [111]. Remarkably, the fraction of flow units needs to overpass the connectivity percolation threshold ∼0.25 before yielding at ∼3% strain, to achieve steady stable flowing. The sample with a faster strain rate relative to the critical strain rate of 2 × 10^−4^ s^−1^ broke before yielding because of the inadequate activated flow unit ratio [75]. Macroscopic flowing beyond 6% strain along with the complete activation of flow units indicates a strain-induced glass transition.

## PROPERTY OPTIMIZATION BASED ON THE FLOW UNIT PERSPECTIVE

### Correlation between flow units and mechanical performance of metallic glasses

**Figure 9.**
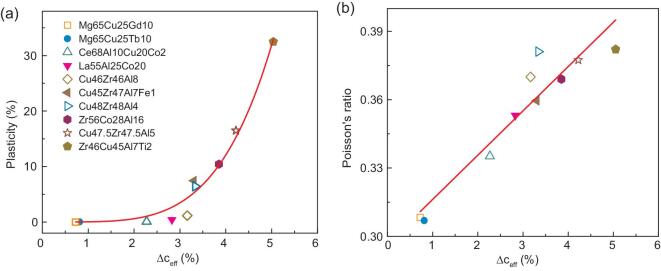
The correlation between (a) plasticity and Δ*c*_eff_ and (b) Poisson's ration and Δ*c*_eff_. Δ*c*_eff_ is the difference of Δ*c*_eff_ (0) among various MG systems; a larger Δ*c*_eff_ means a larger *c*_eff_ (0) value. Data taken from [115].

The characteristics of flow units in various MG systems and the related activation processes are the dominating factors responsible for the diverse mechanical behavior in the operating temperature range. Based on the flow unit perspective, a general property relation in terms of the fraction of flow units in MGs is obtained as [112–114]:

(13) \begin{equation*}P = \frac{{{P_\infty }}}{{1 + {c_{{\rm{eff}}}}(t)}}\end{equation*}

where *c*_eff_ (*t*) is an aging-time-related effective density that is proportional to the intrinsic fraction of the flow units; *P* and *P*_∞_ represent the properties of the real MG and ideal MG samples, respectively. *c*_eff_ (0) is a constant for each composition corresponding to an initial fraction of flow units of as-cast MG. When *c*_eff_ (∞) approaches zero, this corresponds to the ideal MG status without flow units. In practice, the properties after longtime aging are often used as an approximation instead of *P*_∞_. A positive correlation between the compression plasticity and effective density *c*_eff_ (0) of flow units was observed in various MGs [115], as shown in Fig. 9. A system with more intrinsic flow units will exhibit more ductile deformation, because a larger fraction of the activated flow units promotes cooperative motion and multiple shear band formation. Poisson's ratio has been proven to be a successful parameter to predict the plasticity or brittleness of MGs [116]. Figure 9 shows an approximately linear correlation between *c*_eff_ (0) and Poisson's ratio. In general, an MG system with a larger Poisson's ratio has a greater fraction of flow units, and an improved plasticity. A more detailed analysis further found that the bulk/shear modulus softening in flow units also contributes to the change of Poisson's ratio [117]. Mechanical softening could be due to shearing or dilation of flow units, which will lead to a shear band or cavitation. The flow unit perspective considering both processes is closer to the real situation compared to the concept of STZ, where only shear softening provides the mechanism for plasticity instability. This result can also explain the anomalous brittle behavior for some MGs with large Poisson's ratio [117].

Fracture morphology is another practical method to analyze the ductile-to-brittle transition in MGs. Numerous fracture patterns such as dimple structures, periodic corrugations, and river patterns can be found in different regions of a fracture surface, which implies distinct deformation mechanisms for MG systems [118–121]. A typical dimple structure in front of a crack tip and its size distribution characterize the mechanical properties of MGs [118,122]. The dimple size *λ* is proportional to the square of the fracture toughness *K*_c_. This means that a larger dimple structure corresponds to a tougher MG composition [103]. Based on the flow unit perspective combined with a stochastic activation process, the probability distribution of a dimple size can be obtained by the equation [122]

**Figure 10.**
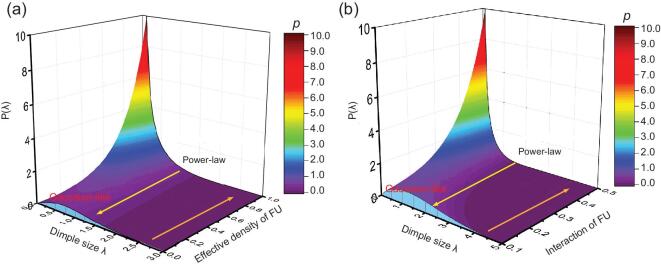
The transition of the dimple distribution from power law to Gaussian-like, which is driven by (a) the density of the flow units and (b) the interaction intensity of the flow units. Adapted with permission from [123], Copyright 2017, Elsevier Ltd.

(14) \begin{equation*} p(\lambda )\,{\lambda ^{ - \beta }}\exp( - 2{I_{\rm FU}} {\lambda ^2}) \end{equation*}

where *I*_FU_ represents the interaction of the flow units and $\beta = 4{I_{{\rm{FU}}}}{c_{{\rm{eff}}}}(0) - 1$ represents the combined effect of the effective density and the interaction of the flow units. From Equation 14, the distribution of the dimple structure *p*(*λ*) will go through a transition from a power-law to a Gaussian-like distribution, with decreasing flow unit density or with a weakening of the interactions among the flow units [123], as shown in Fig. 10. From the experimental data, the as-cast sample with a power-law-distributed dimple structure has a larger average dimple size *λ* compared to an annealed sample with a Gaussian-like distribution [123]. Therefore, the embrittlement in MGs is actually encoded in the evolution of the flow units.

The boson peak is a dynamic anomaly located in the ultrafast THz region, and is a general feature of all types of glasses [124]. This anomaly can also be reflected in an excess specific heat below 40 K. Intensified boson peaks were found to relate to the increment of highly localized nanoscaled shear units [125], implying a connection between this ultrafast dynamics and the mechanical behavior of MGs. A hand-in-hand evolution of the boson heat capacity anomaly and β-relaxation has been observed in recent experiments [126]. These two kinds of fast dynamics should both rise from spatially randomly distributed soft regions of flow units. The boson peak is considered to be associated with the quasi-localized vibrations of atoms embedded in flow units, causing a swing of the system energy within the local basin in the PEL. Heightened or weakened strength of the flow units will alter the state of the boson peak. This physical picture was also supported by the memory effect found in the boson peak [127]. Despite the lack of consensus regarding the origin of the boson peak, this plausible explanation based on flow units provides new insight into the understanding of the relation between atomic motion and mechanical behavior.

**Figure 11.**
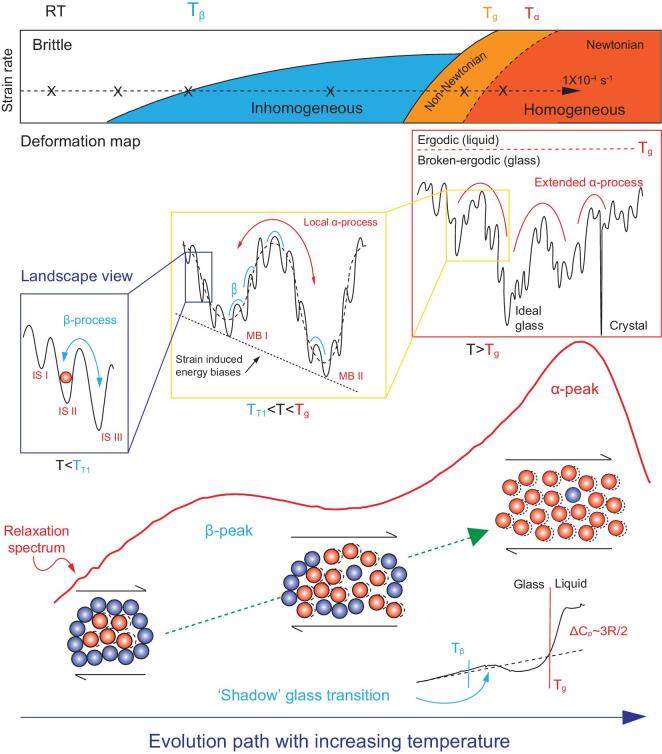
The correlations between the evolution of liquid-like zones, deformation map, relaxation spectrum and energy landscape in MGs. Adapted with permission from [73], Copyright 2014, Nature Publishing Group.

Based on recent results for the flow units, intrinsic correlations between the evolution of flow units, deformation and relaxation maps can be established and summarized for MGs as shown in Fig. 11 [73]. The panorama picture reveals the evolution of localized flow from a low-temperature glassy state to a supercooled liquid state, and facilitates the interpretation of the deformation and relaxation maps and glass-to-liquid transition. Unstable flow units persist in the glassy state, and their reversible activation contributes to the viscoelasticity. The flow units show a nonlinear increase with temperature and their properties play a crucial role in determining diverse flow phenomena in glasses. A connectivity percolation state is achieved above *T*_β_, which leads to ductile deformation and cooperative glass transition processes. This picture sheds light on the mechanism of the flow phenomena in the glassy state and provides a practical guideline in terms of controlling the behavior of metallic glasses. Some case studies based on this idea are presented in the next section.

### Achieving desired performances by tuning the properties of flow units

Based on the flow unit model and its correlation with mechanical properties, optimization of MGs can be achieved by tuning the effective density and activation status of these units. Several cases are listed as examples in the following section.

**Figure 12.**
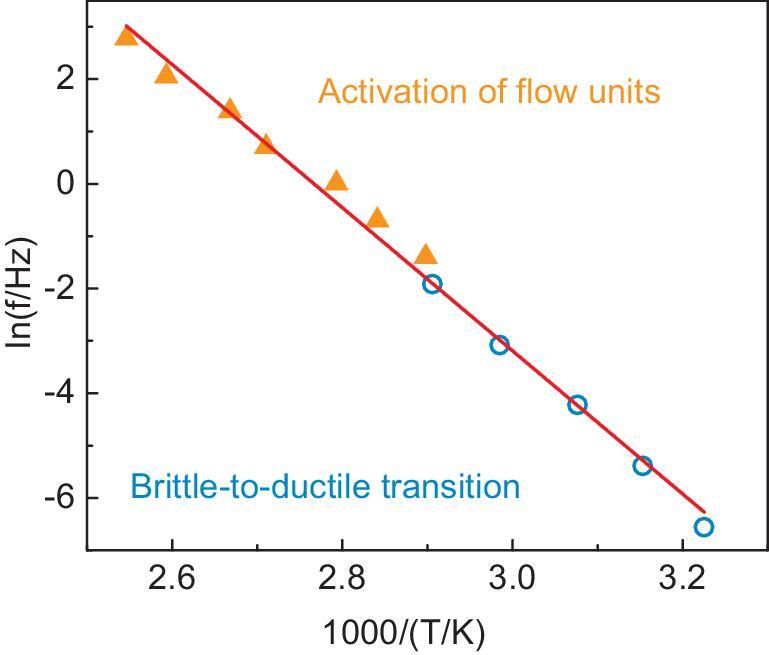
The activation plot of the flow units and brittle-to-ductile transition in both frequency and temperature regimes. Data taken from [128].

By deliberately choosing combinations of elements or micro-alloying elements, local chemical interactions and atomic cluster packing can be altered. This results in the change of fraction and distribution of flow units [62]. The incorporation of 1.5 at.% Co addition into ternary LaNiAl MG and the higher quenching rate tuned the activation of flow units near room temperature, and macroscopic tensile plasticity begins to appear at 313 K with a strain rate of 1 × 10^−5^ s^−1^ [128]. Moreover, brittle-to-ductile transition occurs in the same temperature range and collapses into a single master curve with the activation process of the flow units [128], as shown in Fig. 12. The addition of extra elements and high quenching rate induces a high fraction of flow unit features, which leads to macroscopic plasticity in MGs based on the flow unit perspective.

**Figure 13.**
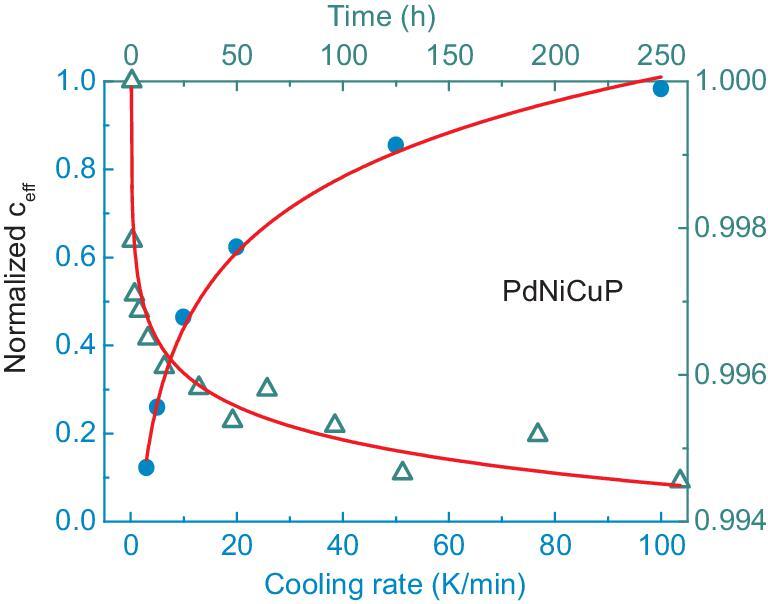
Normalized effective density *c*_eff_ change with different cooling rates and annealing time, which shows an opposite trend related to the properties of MGs. Data taken from [112] and [129].

Changing the location of the system energy state in the PEL causes a variation in the spatial and temporal heterogeneity, and the distribution of intrinsic flow units. MG samples obtained under a slower cooling rate always drop into a deeper basin with a lower energy and become more homogeneous, and *vice versa* [56]. By calculating the enthalpy change of both LaNiAl and PdNiCuP MG samples using differential scanning calorimetry heat-flow curves, a clear increasing trend of the effective density of the flow units can be observed for a faster cooling rate, as shown in Fig. 13 [129]. Almost no flow units exist after the slow cooling process, which agrees well with the observed absence of fast β-relaxation dynamics in ultrastable glass [66]. Annealing the as-cast sample below *T*_g_ is a reverse procedure against cooling, which will lower the MG system energy to a more stable state. Flow units are annihilated with an increasing annealing time, which affects many properties, including hardness, plasticity, modulus and packing fraction [112–114]. The changes in the properties all follow the relationship of Equation 13 and are determined by the flow units.

A strong rejuvenation effect can be introduced in MGs via a low-temperature thermal cycling procedure, which is different from conventional annealing [130]. Tested samples were cycled from room temperature to liquid-nitrogen temperature (77 K), leading to an increase in the overall heat of relaxation. The increase of energy will raise the system location in the PEL and introduce a higher degree of heterogeneity and a higher effective density of the flow units. Enhanced flow unit activity from non-affine thermal strain cycling significantly improves the plasticity of MGs [130]. Besides, mechanical strain annealing by the mandrel winding method is also able to rejuvenate and activate abundant flow units by maintaining the MG sample under a small strain for sufficiently long time (∼24 h) [131,132]. Remarkable homogeneous plastic deformation can be achieved at room temperature by applying this method.

High-pressure annealing was recently found to be an effective and controllable method for tailoring the characteristics of flow units, and can therefore be used to optimize the properties of MGs [133–135]. Bulk PdNiCuP MG with enhanced kinetic stability was formed under 17 GPa at room temperature [133]. Under high pressure, the atoms become densely packed with an increased density, and the annihilation of flow units follows in a similar manner to thermal annealing, but is more effective by pressure. After adjusting the pressure level and further combining with thermal annealing, high energy was successfully stored and preserved in bulk MG samples, and the energy states can be continuously altered using this approach [134,135]. The rejuvenation attributed to the coupling effect of high pressure and high temperature leads to unique structural heterogeneity that contains ‘negative flow units’, with a higher atomic packing density compared to that of the elastic matrix of MGs [134].

## OUTSTANDING ISSUES WITH FLOW UNIT PERSPECTIVE

Although it has proven to be successful in explaining some recent experimental results as discussed in the previous sections, the flow unit perspective is still in its infancy and far from being mature and complete. One main limitation of this approach is that direct identification of these high-mobility flow unit regions is quite difficult in real MG samples. This is because the observation must simultaneously satisfy the requirements of 3D detection on a large scale, atomic-scale spatial resolution and *in situ* recording with high temporal resolution, in accordance with the definition. The lack of explicit *in situ* characterization of flow units means that a robust understanding of the roles played by these regions under external stimulation is yet to be achieved. Currently, the flow unit can only be identified from the dynamics distinction and the direct connection with structural heterogeneity is not clear. Some previous studies, including investigations involving numerical simulations, have determined that the rearrangements of atoms under shear are purely dynamic processes and have no clear correlation with *a priori* structural ‘defects’ [136,137], which cannot be rationalized using the current flow unit model. Besides, by treating the time-dependent features of flow units as mechanical dashpots with a distribution of relaxation time and activation energy, the model can describe the mechanical behavior of MGs under many situations as discussed in the sections entitled ‘Flow unit perspective in metallic glasses’ and ‘Properties of flow units and their evolution under external fields’. However, a more precise mathematical description, especially one that considers the distributed characteristics of the flow units and the interactions between them in a quantitative manner, is not possible without knowledge of these regions that is directly obtained from experimental measurements. In addition, there are also some challenges and unresolved issues in the flow unit perspective. For example, the heterogeneous nature and the system history effect in the current model may be closer to reality, but such assumptions will definitely increase the complexity of numerical simulations. The relation with the glass transition is discussed in the section entitled ‘Evolution processes of flow units under external temperature fields’, where the entire sample is treated as liquid-like above *T*_g_ at the experimental timescale. Thus, this model is rendered unsuitable for studying the phenomena observed in the supercooled liquid state. In regard to property optimization, the validity of the flow unit perspective is currently limited to fully amorphous samples, by tailoring the heterogeneous structure and potential energy state. The connections with many successful practical methods such as the introduction of designed crystalline structures [138,139], martensite phases or twinning structures [140,141], and through surface hardening [142], are not involved. Finally, the flow unit perspective is merely tested and applied to MG systems. It is still unknown whether it can be used for other glassy systems or is unique to MG systems.

## SUMMARY AND OUTLOOK

Featured as dynamic ‘defects’ in MG systems, flow units are considered not merely as a theoretical concept but rather as existing in samples based on the spatial and temporal heterogeneous character of MGs. Flow units are closely connected with the governing of the dynamics of β-relaxation modes in a glassy state. They are responsible for viscoelastic features under stress or strain and behave in a liquid-like manner described by the time-dependent flowing function in MGs. These relatively mobile, thermally and kinetically unstable regions that constitute flow units can be activated by either an external applied temperature or stress input, or both. Cooperative movement of flow units is critical to mechanical performance or otherwise leads to glass transition of MGs. A general property relation controlled by the effective density of flow units is proposed and testified in various MG systems. Desirable MG properties can be designed and optimized using different practical methods through modifications of the characteristics of flow units. Insights gained from the current flow unit perspective will be beneficial to property-oriented MG design. Meanwhile, there are still some open questions that are left for this model. These challenges include *in situ* observations of flow units in both spatial and temporal spaces in MGs, quantitative theoretic descriptions of flow units based on atomic interactions, in-depth understanding of the cooperative motion of flow units and their relation to the glass transition, more predictable flow units and property relationship in the design of desired MG materials, and the possibility of extending the flow unit perspective to other glassy systems.

Before concluding this review, it should be noted that the mechanisms of deformation or flow phenomena in amorphous materials are not thoroughly understood and are far from being complete and unanimous, even in the case of MGs. Discrepancies between theoretical models and the behavior of real samples are well documented. The intended role of flow units is not to replace the classic models, particularly since these models work quite well in many instances and provide useful insights. Instead, this new concept offers a different perspective with regard to its direct applicability to real samples and therefore provides a more bottom-up approach. If we recall the history regarding the discovery of dislocations in crystalline materials, the original concept that considered the elastic fields of defects in homogeneous media was proposed by Volterra in 1907 [143]. Later, in the 1930s, the term ‘dislocations’ was first used by Taylor *et al.* [144] and then widely adopted in the studies related to unsolved questions in crystals. Even though the existence of dislocations was indirectly confirmed via experiments including X-ray analysis, direct observations were not made until the 1950s with the development of transmission electron microscopy [145]. Subsequently, dislocation theory has evolved into an important branch of solid state physics and has proven to be useful in metallurgy [3,146,147]. It took more than half a century to finally identify the dislocations in crystals, which have much simpler configurations compared to glass. ‘History doesn’t repeat itself, but it often rhymes,’ said Mark Twain. The discovery of dynamic defects in glasses has followed a similar track to the identification of dislocations in crystals, and now we are at the precipice of final answers to longstanding questions. We hope that this review will stimulate interest and in-depth investigation in the quest to gain a better understanding of metallic glassy materials and other amorphous systems.
